# Humans Can’t Resist Robot Eyes – Reflexive Cueing With Pseudo-Social Stimuli

**DOI:** 10.3389/frobt.2022.848295

**Published:** 2022-03-23

**Authors:** Linda Onnasch, Eleonora Kostadinova, Paul Schweidler

**Affiliations:** ^1^ Engineering Psychology, Department of Psychology, Humboldt-Universität zu Berlin, Berlin, Germany; ^2^ HFC Human-Factors-Consult GmbH, Berlin, Germany

**Keywords:** human-robot interaction (HRI), gaze-cueing, joint attention, anthropomorphism, robot design, collaborative robot (cobot)

## Abstract

Joint attention is a key mechanism for humans to coordinate their social behavior. Whether and how this mechanism can benefit the interaction with pseudo-social partners such as robots is not well understood. To investigate the potential use of robot eyes as pseudo-social cues that ease attentional shifts we conducted an online study using a modified spatial cueing paradigm. The cue was either a non-social (arrow), a pseudo-social (two versions of an abstract robot eye), or a social stimulus (photographed human eyes) that was presented either paired (e.g. two eyes) or single (e.g. one eye). The latter was varied to separate two assumed triggers of joint attention: the social nature of the stimulus, and the additional spatial information that is conveyed only by paired stimuli. Results support the assumption that pseudo-social stimuli, in our case abstract robot eyes, have the potential to facilitate human-robot interaction as they trigger reflexive cueing. To our surprise, actual social cues did not evoke reflexive shifts in attention. We suspect that the robot eyes elicited the desired effects because they were human-like enough while at the same time being much easier to perceive than human eyes, due to a design with strong contrasts and clean lines. Moreover, results indicate that for reflexive cueing it does not seem to make a difference if the stimulus is presented single or paired. This might be a first indicator that joint attention depends rather on the stimulus’ social nature or familiarity than its spatial expressiveness. Overall, the study suggests that using paired abstract robot eyes might be a good design practice for fostering a positive perception of a robot and to facilitate joint attention as a precursor for coordinated behavior.

## Introduction

In 1969, hundreds of people stood at a corner in New York City staring at the sky. What had happened? Nothing. The scenario was part of an experiment conducted by Milgram and others on the drawing attentional power of crowds (Milgram et al., 1969). Even a single person staring at the sky induced passersby to look up in the direction of the person’s gaze. Although the experiment was mainly interested in the effect of increasing group size on passersby’s attention, it also demonstrated a very powerful underlying mechanism coordinating human behavior: Joint attention, the reflexive directing of one’s attention to an object that another individual is attending to (Shepherd, 2010). This mechanism is key for social intelligence (Alahi et al., 2014). When we observe someone attending to something, we do not only observe the actual behavior of that person but also infer certain mental states, motives and intentions (Baron-Cohen, 1997). This, in turn, enables us to understand, predict and adapt to the situation. Evolutionary, this capability was beneficial as the attentional focus of someone might have indicated the direction of an approaching saber-tooth tiger. The idiosyncratic morphology of the human eye might have even evolved to foster easy discrimination of gaze direction (Kobayashi and Kohshima, 1997), thus facilitating the formation of joint attention and the rise of complex social structures (Emery, 2000; Tomasello et al., 2007). Nowadays, we are not confronted with ancient predators anymore but with new challenges inherent in our increasingly technological environment. Our biological constitution based on these evolutionary developments, however, still remains the same. To ease interaction with new technologies we should therefore strive for a design that exploits our fundamental social mechanisms. In the current study, we addressed the question if joint attention also applies to human-robot interaction (HRI). Robots are increasingly moving into human environments with applications ranging from elderly care to collaborative work in industrial line productions. This implies a growing need to communicate and coordinate with robots. In human-human interaction, joint attention is a precursor for coordinated behavior (joint action; Frischen et al., 2007) and therefore could also support HRI as a profound resource-efficient mechanism (Neider et al., 2010).

Several studies have revealed that the effect of joint attention is only evoked by social stimuli, i.e. eyes. Non-social stimuli, for example, arrows, indicating a specific point of interest also trigger a reallocation of attention, but not as reflexively as social cues. This is revealed by increased reaction times when cues are valid, shorter reaction times when cues are invalid, and an overall smaller gaze cueing effect (Ricciardelli et al., 2002; Friesen et al., 2004; Ristic and Kingstone, 2005). However, which category robots fall into, social or non-social, is not clear and depends largely on a lifelike, specifically anthropomorphic, design of the robot. Anthropomorphism describes the human tendency to attribute humanlike characteristics and behavior to non-human agents (Duffy, 2003). A humanlike robot appearance, e.g., a robot design with legs, arms, and a body, using eyes, mimics, or gestures, fosters this individual tendency, therefore creating a stronger association of robots with social interaction schemes and social categories. In accordance, previous studies have suggested that anthropomorphic robots are more successful than less anthropomorphic robots at conveying “intentions”^1^ through gaze (e.g., Mutlu et al., 2009a; Mutlu et al., 2009b). But even considering anthropomorphism as an explanation for mixed study results in HRI, there is a lack of clear evidence regarding the effectiveness of cueing human attention with robots’ gaze.

For example, Admoni and others (2011) could not find reflexive cueing effects for robotic stimuli, neither for highly nor lowly anthropomorphic designs. The study was conducted using the Posner paradigm (Posner, 1980), i.e. an experimental set-up for spatial cueing. Results showed that, although participants were able to infer directional information from the robot’s gaze, they did not reflexively reorient attention in the direction of the robot’s gaze. In contrast, participants that were presented with human faces (pictured, line-drawn) or arrow stimuli showed the respective attentional shift (Admoni et al., 2011; Admoni and Scassellati, 2012). However, results should be interpreted with caution, because first, it is not clear why social cues did not differ from arrows as non-social cues, second, the study only revealed a main effect of trial validity but no significant effects with regard to stimulus type (nor interaction effects), and third, the statistical power of this study was rather low (included only eight cued trials; Chevalier et al., 2020).

Other studies on robot gaze suggest that robots are perceived as social or at least pseudo-social agents. Chaminade and Okka (2013) again used the Posner paradigm and focused specifically on reflexive cueing mechanisms. The study investigated early processes of social attention orientation with human and with anthropomorphic robot stimuli (Nao robot). Results showed that a robot with anthropomorphic facial cues triggers a reflexive reallocation of attention just as human stimuli do. Interestingly, they observed an increase in reaction times to robot stimuli compared to the human stimuli. This was attributed to the pseudo-social morphology of the anthropomorphic robot. The processing of such ambiguous stimuli might be associated with an increased cognitive effort.

Results by Mutlu and others are also in line with the assumption that robots or robotic stimuli are perceived as (pseudo-)social agents. The study found that physical^2^ robots influenced people’s decisions in a game when the robot shifted its eyes briefly to a certain target (Mutlu et al., 2009b). Moreover, Boucher et al. (Boucher et al., 2012) found that people used a robot’s gaze similarly to human gaze to infer a target position before this position was verbalized by the robot. Also, Wiese and others could show that a robot’s gaze was reliably followed by participants and therefore was capable of establishing joint attention (Wiese et al., 2018). The benefit of perceiving robots as (pseudo-)social agents was furthermore shown by Moon et al. (2014). The study revealed that implementing social gaze in a handover task with an industrial robot improved efficiency. Moreover, participants showed positive attitudes towards this gaze-mediated interaction (Moon et al., 2014).

Although all studies mentioned above primarily focused on gaze effects in HRI, their stimuli always provided more information than only eyes would do. The participants either saw entire faces (Admoni et al., 2011; Admoni and Scassellati, 2012; Boucher et al., 2012; Moon et al., 2014; Mutlu et al., 2009a; Mutlu et al., 2009b; Wiese et al., 2018) or even the robot’s (or human’s) torso (Chaminade and Okka, 2013). The specific importance of eye gaze for joint attention has therefore not been carved out by these studies, as the observed effects might have been confounded by additional informational cues (e.g. head tilt).

Moreover, when comparing (pseudo-)social stimuli to non-social stimuli, there is an often disregarded factor introducing variance: the number of stimuli. Social stimuli are normally presented pairwise, i.e. two eyes, whereas only a single stimulus, typically an arrow, is presented in non-social conditions (Friesen et al., 2004; Admoni et al., 2011; Admoni and Scassellati, 2012). A systematic comparison between paired and single cues is therefore needed to discern whether observed effects are primarily due to differences in the social nature of stimuli or due to the difference in parity. First evidence in favor of the latter interpretation was provided by Symons et al. (2004) in a set of studies comparing the effect of two-eye and one-eye stimuli (full human face, half-human face visible to the observer) on the accuracy in determining the direction of gaze around a target object. Results indicated that information from both eyes was used by observers to determine the direction of gaze as revealed by lower acuity in the one-eye conditions throughout experiments. However, as Symons et al. (2004) only compared human faces, i.e. only social stimuli, it still has to be clarified if results are to be interpreted as an incremental effect where parity merely enhances the effectiveness of social stimuli or if the number of stimuli is the key variable.

The current study therefore aimed at providing specific insights on the effectiveness of social, pseudo-social and non-social “gaze” cueing effects and to disentangle if assumed positive effects of (pseudo-)social cues are mainly due to the social nature and familiarity of cues or to the additional information provided by paired representations. Based on the body of research, we hypothesized that *1*) social stimuli trigger reflexive gaze cueing, *2*) pseudo-social stimuli do as well, but to a lesser extent, and *3*) that non-social stimuli do not elicit reflexive gaze cueing effects at all. Moreover, we explored the effects of paired vs single stimuli to gain insight into the underlying processes of effective reflexive cueing (spatial information vs familiarity of cues). The study’s overall objective was to inform whether and how the beneficial effects of joint attention in human-human interaction (social setup) can be applied to HRI (pseudo-social setup).

## Methods

To ensure transparency and in compliance with good research practice, the study was submitted to and approved by the ethics committee of the Humboldt-Universität zu Berlin. Prior to conducting the experiment, we registered the study at the Open Science Framework (osf.io/ecta9), where also the raw data of the experiment is available.

### Participants

A sample size of *N* = 176 was defined based on an *a priori* power analysis using GPower (Faul et al., 2007, 2009). Accounting for possible exclusions and in order to obtain equal observations across conditions, we recruited 184 German adult native speakers (61 females, M = 29.4 years, SD = 9.40 years) through the crowdsourcing platform Prolific. Participants received 4.20 £ as monetary compensation after the successful completion of the experiment (monetary compensation was aligned with the German minimum wage and converted to GBP as required by prolific).

### Apparatus and Task

**FIGURE 1:**
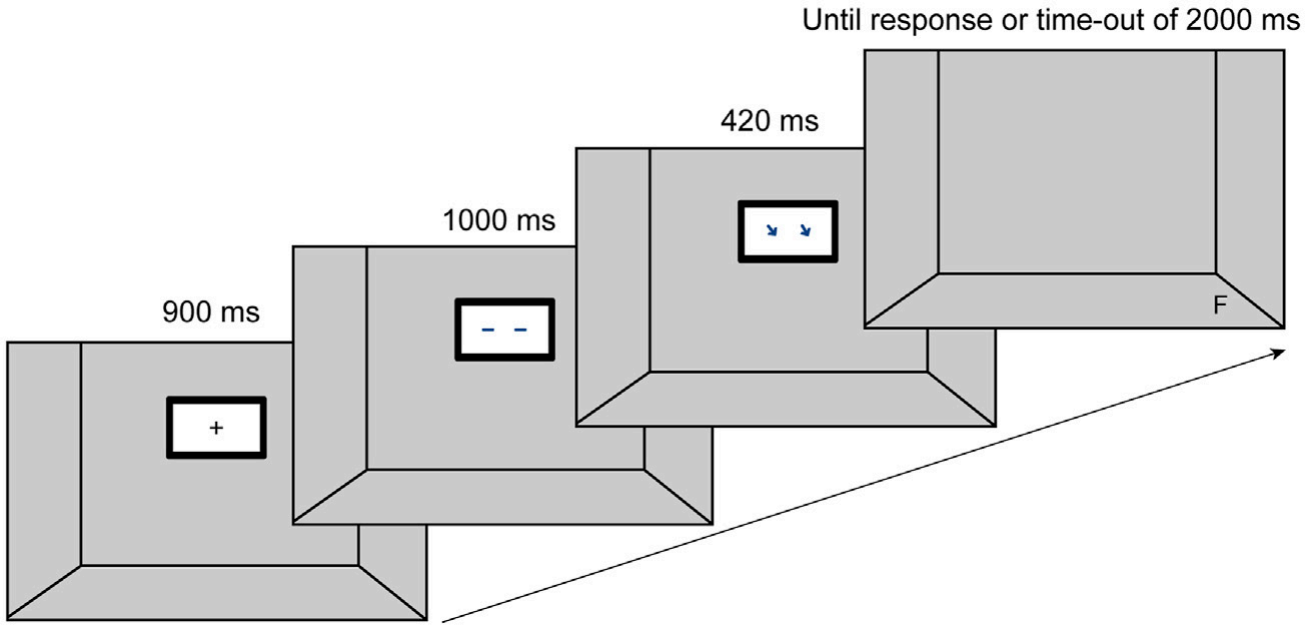
Set-up and sequence of events on a given valid trial. Each trial began with the presentation of a fixation cross in the centre of the “room.” After 900 ms, a display appeared with a “gaze” facing to the front. 1,000 ms later the gaze averted to one of the eight target positions. After a stimulus onset asynchrony (SOA) of 420 ms the target appeared in one of the eight positions. The target disappeared upon participants’ reaction or a time-out of 2000 ms.

To investigate the potential gaze cueing effect of (pseudo-)social stimuli we conducted an online study using a modified version of a traditional spatial cueing paradigm (Posner, 1980; *see* Figure 1). In these setups, participants have to look at a fixation cross, which is then replaced by a spatial cue indicating the position (up, down, left, right) of a subsequently following target stimulus to which participants have to react by an according key press as fast as possible. Our modifications to the traditional setup included: *1*) the use of a three-dimensional-like space, within which the cueing stimuli appeared on a depicted display, and *2*) the use of eight target positions, rather than the standard two or four. In modulating the task in the described way, we sought to recreate an experimental setting with a higher ecological validity for the use of a collaborative robot (Sawyer) in industrial settings. In such environments, humans typically interact with a robot in a shared space like a worktop at which human and robot have to coordinate their behavior and movements in terms of handovers, or shared actions on a production piece.

To model a three-dimensional-like space with positions corresponding to potential target positions in an industrial HRI we first created a physical setup of a shared workspace with an industrial robot (Sawyer by Rethink Robotics) where we measured the distances between target positions, the robot’s display, and the human co-worker. These distances were then scaled down and transferred as parameters into our model, that used HTML, JavaScript and raster graphics to render the virtual set-up.

Targets were represented by F and T capital letters. Two of the target positions were on the left and right “wall,” peripheral to the display. The other six were placed in front of the display on what would be the shared workspace. Three of the target positions were situated closer to the back of the virtual room, i.e. with more distance to the human, while the other three were placed closer to the human, i.e. more at the front of the room/workspace. No target positions were located above the display since such robot movements are unlikely to occur in close collaboration with humans due to safety regulations.

The cueing stimuli were either images of human eyes, arrows, or two different versions of robot eyes (“pixel,” “crosshair”). All images, except for the human eyes, were created with Adobe Illustrator by professional motion designers (whydobirds). The human eyes were photographs of a male human’s head that were cropped and adjusted to fit our set-up using GIMP (The GIMP Development Team, 2019).

We developed our gaze cueing paradigm using jsPsych, a tool for creating web-based experiments (de Leeuw, 2015). We used jsPsych’s html-keyboard-response plugin to display the content of the experiment and record participants’ responses and reaction times. The study was run in a web browser. To ensure a proper functionality of the set-up, participation required a machine with a keyboard and a minimal browser window resolution of 1,280 × 578 pixels (i.e. no tablets or phones). We further used the jspsych-resize plugin 3 to perform calibration. This allowed stimuli to retain a known, pre-determined size across different monitor resolutions.

### Design

**FIGURE 2:**
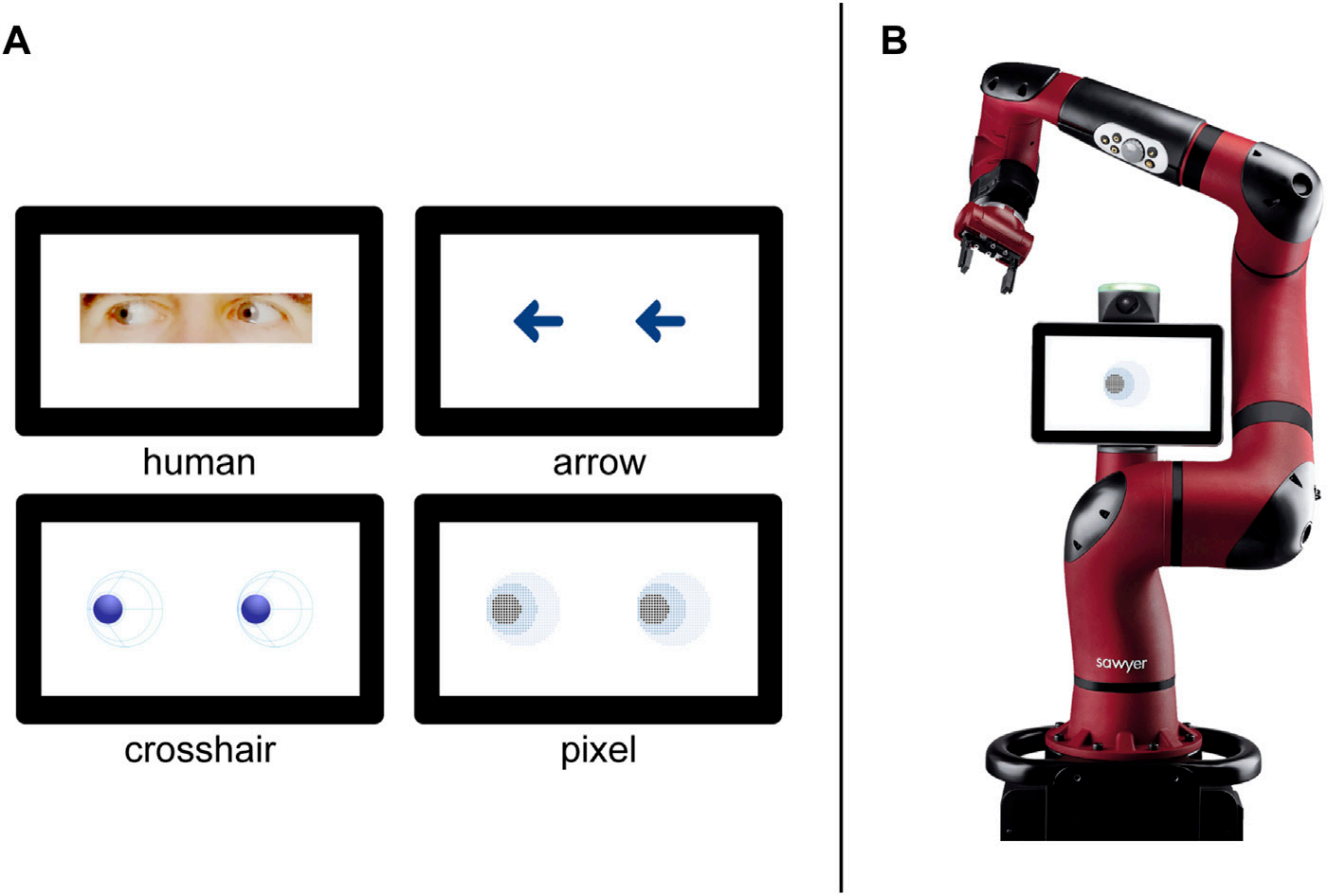
**(A)** The four stimulus types, labelled respectively. Only the paired version is presented here. The top row includes the social (left) and non-social (right) stimuli. The abstract robot eyes are presented in the second row. **(B)** Image of the collaborative robot Sawyer used in the questionnaire. Here, presented featuring the single pixel eye design.

Three variables were systematically varied in our experiment. First, the cues were varied between-subject, representing either, social (human eyes), pseudo-social (robot eyes) or non-social (arrows) stimuli (Figure 2A). The objective in designing the pseudo-social stimuli was to achieve a maximum level of abstraction while retaining the essential features of the human eye (e.g. visible pupil-sclera size ratio). This resulted in two different robot eye designs, that were both exploratively compared in the experiment. The first robot eye design, the pixel design, is an abstract pixelated representation of sclera, iris and pupil. The second robot eye design is more artificial as the figurative idea was based on a crosshair. The representation only depicts the pupil and separates the sclera with an additional circular line and four lines converging on the pupil (both designs are depicted in Figure 2A, bottom row). Second, we varied the number of stimuli between-subject, presenting either single or paired cues to participants. Third, the trial validity was manipulated as a within-subject factor. From a total of 160 trials, the target stimuli appeared at cued locations in 80% of the trials (valid), while in the remaining 20% of trials the target appeared at uncued locations (invalid). Overall, this resulted in a 4 (stimulus type) × 2 (number of stimuli) × 2 (trial validity) mixed design.

### Dependent Measures

#### Control Variables

We included the Affinity for Technology Interaction scale (ATI, nine items, six-point Likert scale) by Franke et al. (2019) and asked participants for previous experience with robots (single item, yes/no, if yes: What kind of experience) to control for possible systematic biases between the different experimental groups. Whereas we did not assume that such differences would affect reaction times in the spatial cueing paradigm, they would have been relevant for measures of the subjective perception of robots.

#### Reflexive Cueing

To evaluate the potential for reflexive cueing of the different stimuli, we assessed the reaction times (ms), measured from the target onset to a key press (F or T) to the target. We only included trials with correct answers (e.g. target F, key press F) as incorrect answers might have biased the results. Additionally, we calculated the gaze cueing effect (GCE) by subtracting mean reaction times of valid trials from the mean reaction times of invalid trials.

#### Additional Exploratory Variables for Task-Related and Social Attributions

Moreover, we were interested in the perception of a robot having incorporated the stimuli that we used in our study. A positive perception would be key in terms of acceptance if such robot designs were to be implemented. Ratings for the following questionnaires were therefore collected by presenting an illustration of the respective stimuli (human eyes, robot eyes, arrows; single vs paired) on the display of an industrial collaborative robot (Sawyer, Rethink Robotics, *see* Figure 2B). First, participants had to answer three single items with regard to the perceived task-related functionality of the stimuli. Questions asked for *1*) perceived accuracy (*I could easily tell where the [stimuli] were pointing at*), *2*) perceived surveillance (*I felt watched by the [stimuli]*), and *3*) perceived usefulness^3^ (*I think the [stimuli] were helpful for potentially identifying the direction of the robot’s arm movement*) on a seven-point Likert scale with semantic anchors (not at all; a lot).

To check the social perception of our stimuli we used three different measures. The first was a questionnaire on mind perception with five items that had to be answered on a seven-point rating scale ranging from “definitely not alive” to “definitely alive” (Martini et al., 2016). Second, we used the Godspeed revised questionnaire (Ho and MacDorman, 2010). This comprises three subscales (humanness, eeriness, attractiveness) with a total of 18 items that had to be answered on a five-point Likert scale. Third, we further asked participants to answer the Robotic Social Attributes Scale (RoSAS; Carpinella et al., 2017). The RoSAS consists of three subcategories (warmth, competence, discomfort) and a total of 18 adjectives. Participants indicated on a nine-point Likert scale from 1 (*definitely not associated*) to 9 (*definitely associated*) how closely each adjective was associated with the robot image.

In addition, participants were asked to indicate how stimulating they perceived the robot designs in terms of hedonic quality. For this evaluation, the according subscale of the AttrakDiff (Hassenzahl et al., 2003) was used which consists of seven items. Answers were provided on a semantic differential with seven gradations.

### Procedure

Participants completed the online study using their own devices. At the beginning, participants received detailed information about the study and data handling. After giving their informed consent, they received instructions for the experiment and started with two trainings that familiarized them with the task. The first training comprised 12 trials during which a letter (T or F) appeared centrally on the screen. Participants were instructed to place their left index finger on the F key and their right index finger on the T key and react upon seeing the letters, using the respective keys on their keyboard. The letter changed its color from white to green upon correct response and from white to red, indicating an incorrect reaction. The aim of this training was to get participants used to the key presses without having to shift their gaze to the keyboard. Participants were offered to repeat the training in case they felt insecure. During the 10 trials of the second training participants practiced the experimental task. They were told they would look into a room in which a display was hanging at the back wall (*see* Figure 1). The appearance of a fixation cross started a trial. Participants had to direct their gaze to the fixation cross, which was replaced after 900 ms by a cue stimulus facing forward for 1,000 ms. Next, the cue stimulus changed “gaze” direction (or pointing direction in case of arrows) towards one of eight possible locations. The cue disappeared after a stimulus onset asynchrony (SOA) of 420 ms, then the target letter was displayed. The target remained on the screen until a response was given or a time-out of 2000 ms was reached. After each trial we integrated an inter-trial interval of 200 ms before the next trial began. After completing the second training, the main test procedure started consisting of 160 trials that lasted five to 7 min in total. The time course per trial as applied to the second training and the experimental trial is shown in Figure 1.

Upon successful completion of the actual experiment, in a last step participants were asked to fill in the remaining questionnaires (mind perception, Godspeed revised, RoSAS, AttrakDiff, ATI, previous experience with robots). The entire experimental procedure lasted approximately 30 min.

### Data Analysis

Reaction times were analyzed with a 4 (stimulus type) × 2 (number of stimuli) × 2 (trial validity) ANOVA with repeated measures. GCE as well as the questionnaires for manipulation check, control variables and the perception of stimuli were investigated with 4 (stimulus type) × 2 (number of stimuli) ANOVAs. The only exception was the dependent variable that asked for participants’ previous experience with robots that was analyzed with a chi-square goodness-of-fit test. For *post hoc* pairwise comparisons, *p*-values were Bonferroni corrected for multiple comparisons.

## Results

### Control Variables

Results for the *ATI* (Franke et al., 2019) revealed no differences between groups (main effects stimulus type, number of stimuli, interaction effect: *F* < 1). With regard to *robot experience*, 36 participants (19.6%) indicated to have had previous experience with robots (mostly with industrial robots because of work, robot vacuum cleaners at home or just seen robots before). The distribution of these participants to the single groups (stimulus type × number of stimuli) did not differ significantly, (χ^2^(7) = 13.33, *p* = 0.064).

### Reflexive Cueing

**FIGURE 3:**
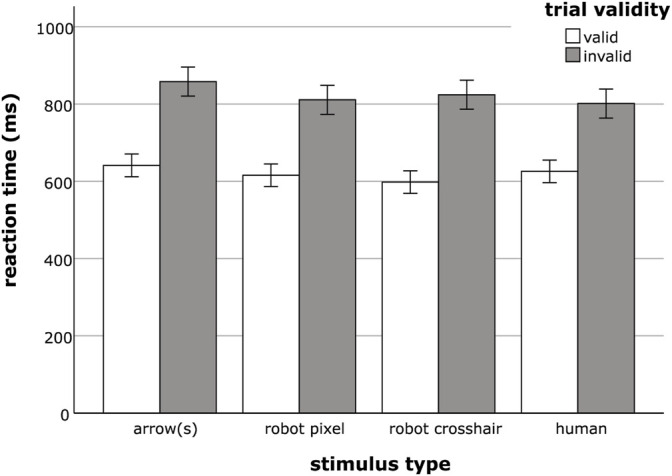
Mean reaction times for valid and invalid trials for the different stimulus type conditions arrow(s), robot pixel, robot crosshair and human. Error bars represent the standard error of the means.

*Reaction times* were substantially slower when the presented cues were invalid (*M* = 823.56 ms; *SE* = 9.53 ms) compared to reactions to valid cues (*M* = 620.06 ms; *SE* = 7.43 ms). This was statistically confirmed by a significant main effect of trial validity (*F*(1, 176) = 1,009.44, *p <* 0.001, $\eta_{p}^{2}$ = 0.85). Moreover, there was a significant interaction between trial validity and stimulus type, as illustrated in Figure 3 (*F*(3, 176) = 3.14, *p =* 0.027, $\eta_{p}^{2}$ = 0.05). In valid trial conditions, participants reacted fastest to the pseudo-social stimuli, i.e. the robot eyes, followed by human eye cues and arrows. When cues pointed to an incorrect target position however, the human eye stimuli led to the fastest responses, followed by the pseudo-social stimuli (both robot eye designs). Slowest reactions were again revealed for the arrow condition.

There were no significant main effects of neither number of stimuli (*F*(1, 176) = 0.52, *p* = 0.471, $\eta_{p}^{2}$ = 0.003) nor stimulus type (*F*(3, 176) = 1.37, *p* < 0.252, $\eta_{p}^{2}$ = 0.02). In addition, no further interactions reached significance (number of stimuli × trial validity: *F*(1, 176) = 1.37, *p* = 0.243, $\eta_{p}^{2}$ <0.01; stimulus type × number of stimuli: *F <* 1; stimulus type × number of stimuli x trial validity: *F*(3, 176) = 0.71, *p* = 0.549, $\eta_{p}^{2}$ <0.01).

**FIGURE 4:**
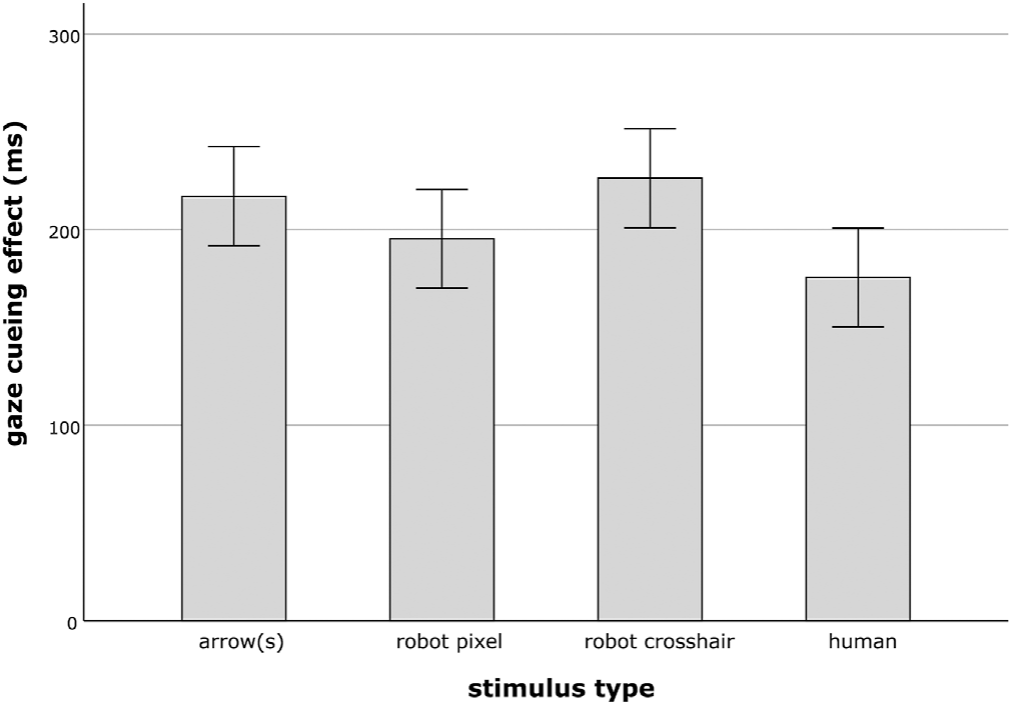
Mean gaze cueing effect of the different stimulus type conditions arrow(s), robot pixel, robot crosshair and human. Error bars represent the standard error of the means.

The 4 × 2 ANOVA on *GCE*s, with stimulus type and number of stimuli as independent variables revealed a main effect of type (*F*(3, 176) = 3.14, *p* = 0.027, $\eta_{p}^{2}$ = 0.05). As Figure 4 illustrates, people showed the largest GCE when cued by the robot crosshair stimuli, followed by the arrow and the robot pixel stimulus condition. The smallest GCE was found for the human eye group. *Post hoc* tests indicated significant differences only when comparing the human stimuli with the robot crosshair stimuli (*p =* 0.034), but not compared to the arrow stimuli (*p =* 0.140), nor the second robot design, the pixel stimuli (*p =* 1).

There was no main effect with respect to the number of stimuli (*F*(1, 176) = 1.37, *p* = 0.243, $\eta_{p}^{2}$ = 0.008), nor was there an interaction between type and number (*F* < 1).

### Task-Related and Social Attributions

**FIGURE 5:**
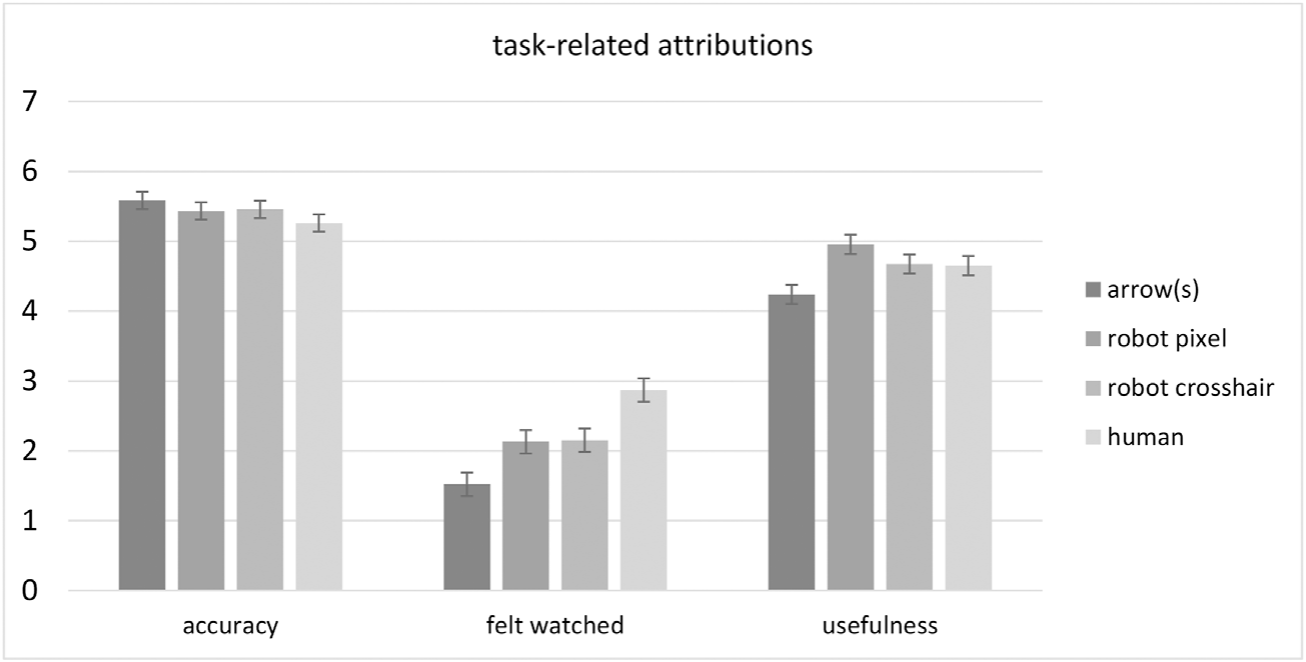
Mean ratings for the three single items addressing task-related attributions (perceived accuracy, surveillance and usefulness) for the different stimulus type conditions arrow(s), robot pixel, robot crosshair and human. Error bars represent the standard error of the means.

Results for the three customized single items are illustrated in Figure 5 and reported in the following. The *perceived accuracy* of stimuli was overall rated as good (*M* = 5.43, *SE* = 0.06) and no significant differences became apparent between groups with respect to stimulus type (*F*(3, 176) = 1.16, *p* = 0.325, $\eta_{p}^{2}$ = 0.01), number of stimuli (*F* < 1), nor was an interaction of both factors revealed (*F*(3, 176) = 1.47, *p* = 0.222, $\eta_{p}^{2}$ = 0.02).

Conversely, responses to the question whether participants *felt watched* by the stimuli were overall low (*M* = 2.16, *SE* = 0.08). However, people felt significantly more watched by human eyes than by robot eyes and arrows. This was statistically supported by a significant main effect of stimulus type (*F*(3, 176) = 10.78, *p* < 0.001, $\eta_{p}^{2}$ = 15) and according *post hoc* comparisons (*p*_*human–crosshair*_ = 0.017, *p*_*human–pixel*_ = 0.013, *p*_*human–arrow*_ < 0.001). No other effects were observed (number of stimuli: *F* < 1; interaction: *F*(3, 176) = 1.41, *p* = 0.241, $\eta_{p}^{2}$ = 0.02).

The last customized question addressed the *perceived usefulness* of the stimuli. Again, there was a difference between stimulus types (*F*(3, 176) = 4.61, *p* = 0.004, $\eta_{p}^{2}$ = 0.07) but not between number of stimuli (*F* < 1), nor was there an interaction effect (*F* < 1). The robot eyes, the pixel design in particular, were perceived as most helpful in potentially identifying the direction of a robot’s arm movement. The arrows as indicators were rated least helpful. *Post hoc* comparisons further detailed this effect and showed a significant difference between the arrow and the robot pixel design (*p* = 0.002).

Results for *mind perception* (Martini et al., 2016) showed a significant effect of stimulus type, *F*(3, 176) = 3.16, *p =* 0.026, $\eta_{p}^{2}$ = 0.05. *Post hoc* tests further detailed this result and showed that only the robot pixel stimuli had significantly higher ratings with regard to mind perception (*M*_*pixel*_ = 2.45, *SE*_*pixel*_ = 0.14) than the arrow stimuli (*M*_*arrow*_ = 1.86, *SE*_*arrow*_ = 0.14; *p =* 0.023). There was no difference in mind perception between arrow and the robot crosshair stimuli (*M*_*crosshair*_ = 2.30, *SE*_*crosshair*_ = 0.14; *p =* 0.166), and surprisingly also not between arrow and human stimuli (*M*_*human*_ = 2.14, *SE*_*human*_ = 0.14; *p =* 0.939). If stimuli were presented in a paired or single fashion did not make a difference with regard to mind perception, *F*(1, 176) = 2.09, *p* = 0.149, $\eta_{p}^{2}$ = 0.01, nor did the data reveal an interaction between stimulus type and the presentation as single or paired cues, *F*(3, 176) = 1.10, *p* = 0.349, $\eta_{p}^{2}$ = 0.01.

Ratings of the Godspeed revised *humanness* dimension (Ho and MacDorman, 2010) were overall relatively low (*M* = 1.90, *SE* = 0.04) and very similar across conditions. The 2 × 2 ANOVA showed neither a main effect of stimulus type, *F*(3, 176) = 0.67, *p* = 0.572, $\eta_{p}^{2}$ = 0.01, nor one of number, *F*(1, 176) = 1.64, *p* = 0.202, $\eta_{p}^{2}$ = 0.09, neither was there a significant interaction, *F*(3, 176) = 1.01, *p* = 0.390, $\eta_{p}^{2}$ = 0.01.

Results for the subscale *eeriness* revealed that human eyes implemented at the robot’s display were perceived as most eerie (*M*_*human*_ = 2.98, *SE*_*human*_ = 0.07) compared to a pixel design (*M*_*pixel*_ = 2.74, *SE*_*pixel*_ = 0.07), a crosshair design (*M*_*crosshair*_ = 2.62, *SE*_*crosshair*_ = 0.07) and an arrow design (*M*_*arrow*_ = 2.63, *SE*_*arrow*_ = 0.07). This was supported by a significant main effect of stimulus type, *F*(3, 176) = 5.45, *p* = 0.001, $\eta_{p}^{2}$ = 0.08. *Post hoc* comparisons revealed significant differences between the human eye design and all other designs (*p*_*human–pixel*_ = 0.021, *p*_*human–crosshair*_ = 0.001, *p*_*human–arrow*_ = 0.001). However, it is noteworthy that although the designs evoked different perceptions, they all scored in the middle range of the scale and even the human eyes still revealed relatively moderate ratings. In addition to the stimulus type, the number of stimuli had a significant effect on the perception of eeriness, too (*F*(1, 176) = 5.99, *p* = 0.015, $\eta_{p}^{2}$ = 0.03). Single stimuli presentations were rated more eerie (*M*_*single*_ = 2.83, *SE*_*single*_ = 0.05) than paired presentations (*M*_*paired*_ = 2.65, *SE*_*paired*_ = 0.05). Data did not reveal an interaction, *F*(3, 176) = 2.06, *p* = 0.105, $\eta_{p}^{2}$ = 0.03.

Mirroring results regarding eeriness, participants rated the robot presented with human eye design as least *attractive* on the Godspeed revised scale (*M*_*human*_ = 2.83, *SE*_*human*_ = 0.09) compared to the other stimulus designs (*M*_*pixel*_ = 3.31, *SE*_*pixel*_ = 0.09; *M*_*crosshair*_ = 3.31, *SE*_*crosshair*_ = 0.09; *M*_*arrow*_ = 3.24, *SE*_*arrow*_ = 0.09). This difference was statistically confirmed by a significant main effect of stimulus type (*F*(3, 176) = 5.51, *p* = 0.001, $\eta_{p}^{2}$ = 0.08) and according *post hoc* comparisons (*p*_*human–pixel*_ = 0.004, *p*_*human–crosshair*_ = 0.004, *p*_*human–arrow*_ = 0.023). No other effects were revealed by the data (number of stimuli: *F* < 1; interaction: *F*(3, 176) = 1.07, *p* = 0.362, $\eta_{p}^{2}$ = 0.01).

On the *warmth* dimension of the RoSAS scale (Carpinella et al., 2017), participants rated the robot crosshair design highest (*M*_*crosshair*_ = 2.95, *SE*_*crosshair*_ = 0.15) while arrows received the overall lowest ratings (*M*_*arrow*_ = 1.94, *SE*_*arrow*_ = 0.15). Accordingly, the 2 × 2 ANOVA showed a significant main effect of type (*F*(3, 176) = 3.95, *p* = 0.009, $\eta_{p}^{2}$ = 0.06) that was further detailed by a significant *post hoc* comparison between crosshair stimuli and arrow stimuli (*p* = 0.019). No significant main effect of stimulus number (*F*(1, 176) = 2.27, *p* = 0.134, $\eta_{p}^{2}$ = 0.01) nor an interaction emerged (*F*(3, 176) = 1.27, *p* = 0.285, $\eta_{p}^{2}$ = 0.02).

Regarding the perceived *competence* of a robot with different stimulus designs (stimulus type, number of stimuli), there were substantial differences that led to a significant interaction effect, *F*(3, 176) = 3.73, *p* = 0.012, $\eta_{p}^{2}$ = 0.06. This was specifically due to the ratings of the pixel design. While for the arrow, the human and the crosshair design a paired stimulus presentation was perceived as more competent, this was reversed for the pixel design. In the latter case, a single presentation was favored over a paired presentation (*see* Table 1).

**TABLE 1:** Mean ratings (and SE) for the perceived competence scale of the RoSAS, differentiated for stimulus type and number of stimuli.

Stimulus type	Single presentation		Paired presentation	
	*M*	*SE*	*M*	*SE*
human	4.68	0.20	4.92	0.20
crosshair	4.66	0.20	5.05	0.20
pixel	5.29	0.20	4.45	0.20
arrow	4.91	0.20	5.09	0.20

Moreover, we investigated the experienced *discomfort* evoked by the different robot designs. The type of stimulus revealed a significant main effect (*F*(3, 176) = 7.77, *p* < 0.001, $\eta_{p}^{2}$ = 0.11) while the number of stimuli had no effect (*F*(1, 176) = 2.21, *p* = 0.139, $\eta_{p}^{2}$ = 0.01) nor was there an interaction between both factors (*F* < 1). Supporting results for eeriness, implementing human eyes on a robot’s display triggered substantially higher discomfort (*M*_*human*_ = 3.24, *SE*_*human*_ = 0.15) than the pixel design (*M*_*pixel*_ = 2.56, *SE*_*pixel*_ = 0.15), the crosshair design (*M*_*crosshair*_ = 2.38, *SE*_*crosshair*_ = 0.15) or the arrows (*M*_*arrow*_ = 2.25, *SE*_*arrow*_ = 0.15). This was statistically supported by *post hoc* comparisons (*p*_*human–pixel*_ = 0.016, *p*_*human–crosshair*_ = 0.001, *p*_*human–arrow*_ < 0.001).

On the *hedonic quality - stimulation* dimension stimuli received higher ratings in the single version compared to the paired version, with the most pronounced difference for pixel stimuli (Table 2). The only exception was the crosshair stimulus, that received higher ratings as a paired design than as a single stimulus. The 4 × 2 ANOVA showed a significant main effect of number of stimuli (*F*(1, 176) = 5.43, *p* = 0:048, $\eta_{p}^{2}$ = 0.03) and a significant interaction between type and number of stimuli (*F*(3, 176) = 2.83, *p* = 0.040, $\eta_{p}^{2}$ = 0.04). No main effect of stimulus type was found (*F*(3, 176) = 2.35, *p* = 0.073, $\eta_{p}^{2}$ = 0.03).

**TABLE 2:** Mean ratings (and SE) for the hedonic quality - stimulation dimension of the AttrakDiff, differentiated for stimulus type and number of stimuli.

Stimulus type	Single presentation		Paired presentation	
	*M*	*SE*	*M*	*SE*
human	4.60	0.19	4.23	0.19
crosshair	3.93	0.19	4.24	0.19
pixel	4.67	0.19	3.87	0.19
arrow	4.14	0.19	3.74	0.19

## Discussion

This study investigated the effectiveness of social, pseudo-social and non-social “gaze” cueing effects and aimed at disentangling whether assumed positive effects of (pseudo-)social cues are mainly due to the social nature and familiarity of cues or to the additional information provided by paired representations. We therefore conducted an online-experiment using a modified version of Posner’s spatial cueing paradigm. To increase the ecological validity we aligned the experimental target cue positions with possible target positions in a physical shared workspace from an industrial HRI setting. We furthermore raised the number of target cue positions to eight (instead of traditional two or four) but limited them to positions below the human’s head as this is a substantial safety requirement in industrial HRI. The increase in complexity resulted in overall higher reaction times and substantial differences between valid and invalid trials compared to previous research (e.g. Chaminade and Okka, 2013; Wiese et al., 2018) indicating overall higher attentional demands of the modified paradigm.

Our main focus of interest was on the effectiveness of reflexive cueing with regard to the different stimulus designs and the number of stimuli. We found a main effect of trial validity. When the presented cues were valid, reaction times to the target stimulus were substantially shorter than compared to invalid cues. This is a common finding in research applying spatial cueing paradigms (Ricciardelli et al., 2002; Friesen et al., 2004; Ristic and Kingstone, 2005). More interesting was the finding of an interaction effect of trial validity and stimulus design. When a cue was valid, the fastest reactions were found for the pseudo-social stimuli, followed by social and then the non-social cues. This pattern is against our hypothesis that social stimuli should trigger the fastest responses. However, it supports the assumption that robot eyes might have been processed as social cues. This interpretation is in line with previous evidence for a triggering effect of robot stimuli comparable to social, i.e., human stimuli (Mutlu et al., 2009a; Mutlu et al., 2009b; Boucher et al., 2012; Chaminade and Okka, 2013; Moon et al., 2014; Wiese et al., 2018). The slower reactions to the human stimuli might have been due to a lack of saliency compared to the other stimuli. Although the overall image size was the same in all conditions, the human eyes were smaller and less rich in contrast than the other cues. Yet, if it was only high contrast imagery that led to a shorter processing time, then the chunky and clearly defined, but non-social arrow stimuli should have triggered the fastest reactions or at least comparable results to the pseudo-social robot stimuli. Since they did not, results speak for the primacy of social processing of the robot stimuli.

In a similar line of thought, human stimuli inspected in most screen-based paradigms are schematic faces or eyes (e.g. Friesen and Kingstone, 1998). One might argue that our pseudo-social robot stimuli more closely resembled these schematic human eyes. The strong cueing effects of our robot stimuli then should not be surprising. In turn, this might question the validity of previous studies using such stimuli as the social, i.e. human cues. Although these stimuli are essentially coined social and biological, they are stripped out of their biological realism and, importantly, their social relevance (discussed in a review by Dalmaso et al., 2020). However, the latter might also apply to the social cues used in the current study. Although we presented real human eyes, these were still images that were additionally embedded in a robot display. It is therefore conceivable that the human eyes were not interpreted as actual eyes, but as images of such. This differentiation is important as gaze cueing effects are not purely reflexive (bottom-up), but can be modulated by top-down cognitive processes like social context information of the observed scene (Wiese et al., 2012). If the human eye stimuli were interpreted as images instead of an actual social cue this might have disrupted the social processing to a certain extent. Support for this is provided by our results in invalid cueing trials, in which the human eye stimuli led to the fastest responses. This was also mirrored in the GCE that was smallest in the human eye group and largest for one of the pseudo-social stimuli, the robot crosshair condition. Future studies should therefore strive to ensure the perception of human eyes as truly social and intentional. This could be done, for example, by appropriate framing in which participants are told that stimulus movements are controlled online by a human model (e.g. Wiese et al., 2012) or by using real embodied stimuli, e.g., in the form of videos.

Another notable observation from our study relates to the use of a single-stimulus interface. With the comparison of paired and single stimuli we wanted to explore whether a faster and more accurate cueing effect of (pseudo-)social stimuli is mainly due to the social nature of stimuli or to the additional spatial information that is provided by two stimuli. In contrast to results by Symons et al. (2004) suggesting that information from both eyes was used by observers to determine the direction of gaze, we could not find substantial differences in the reaction time to single or paired stimuli. Results from the current study therefore speak in favor of the social aspect as the key for preferential processing of information and consequently faster responses. As this was to the authors’ knowledge the first study that systematically varied this aspect in the context of gaze cueing efficiency comparing social and non-social cues, future studies are needed to replicate findings and further inform about the underlying effects of faster responses to such single and paired cues. If this effect proves valid, single cue interfaces might be a design solution for robots whose task or context do not favor high human-likeness.

In addition to investigating the general effectiveness of reflexive cueing potentials of the different stimuli, we were interested in the perception of a robot in which these stimuli were integrated. A positive perception would be key for acceptance if such robot designs were to be implemented. We therefore asked how participants perceived the different designs as a robot’s interface. Results revealed that the perceived accuracy of all stimuli was overall good. However, people felt they were being watched by the human eyes whereas the robot stimuli were rated as being most helpful in predicting robot arm movements.

With regard to social attributes, the study revealed an overall favorable perception of the robot stimuli, too. The robot pixel eye design induced higher perceptions of mind compared to clearly non-social stimuli, the arrows. Surprisingly, human eyes as well as the robotic crosshair design did not differ from arrows in terms of mind perception. These somewhat unexpected results might be due to the fact that we presented an illustration of the stimuli on the display of an industrial collaborative robot. In this clearly technical overall appearance, human eyes did not fit to the rest of the robot, which might have evoked a rather eerie overall design impression instead of supporting a social and lifelike perception of the robot. This interpretation was supported by results of the eeriness subscale of the Godspeed revised questionnaire, as well as the discomfort scale of the RoSAS, that both revealed substantially higher ratings for the human eyes compared to all other stimuli. These findings might be explained by the matching hypothesis (Berscheid et al., 1971). Originally formulated in the context of social psychology and human-human relations, the hypothesis also seems to apply to preference perceptions in HRI (Goetz et al., 2003; Klüber and Onnasch, 2022). Robots are preferred either if *1*) appearance matches the task that should be performed or *2*) that present an overall coherent image. With regard to the stimuli of the current study, we assume that the pseudo-social stimuli had the highest match to the overall appearance of the robot that was presented.

The results of the Godspeed revised humanness scale were overall relatively low and revealed no differences with regard to stimulus type or number of stimuli. The low ratings with regard to the pseudo-social stimuli were as expected, as we explicitly aimed at a stimulus design that used as few anthropomorphic aspects as possible, and that only transferred the functional qualities of human eyes to the pseudo-social stimuli while avoiding a too human-like design. This approach was based on previous studies, showing that an anthropomorphic design in an industrial context (which applied here as well) is detrimental to trust and perceived reliability of the robot (Roesler et al., 2020; Roesler et al., 2021; Onnasch and Hildebrandt, 2022). Support for these findings was also provided by the results for the implemented human eyes in the current study. While these were still not rated very human-like in the overall robot design, they induced perceptions of eeriness and discomfort that would most likely decrease the acceptance and use of an actual robot incorporating such a design.

Furthermore, the robot eye designs were rated highest for warmth, were not discomforting nor eerie and did not differ from the other designs in terms of attributed competence. An interesting effect, however, was that whereas for all designs the paired stimulus presentation was perceived as being more competent, this was reversed for the pixel pseudo-social stimuli. In this case, a robot displaying a single stimulus was rated as being more competent. Since this effect is lacking a theoretical underpinning, it might just be a sample bias and should therefore not be given too much importance (unless replicated).

Last but not least, we were interested in the hedonic quality of our chosen stimulus designs. Hedonic quality aims to capture the experience that is not related to instrumental aspects of a system but the sensual experience and the extent to which this experience fits individual goals (Rafaeli and Vilnai-Yvetz, 2004). It represents the need for novelty and variety as well as inspiration (Hassenzahl, 2001). Having these attributes in mind, it is not surprising that the most unusual stimulus designs (e.g. single stimuli) scored best on this variable. Single stimuli were favored over paired ones. The only exception was for the pseudo-social crosshair stimulus design which had higher ratings in the paired condition. This makes it an interesting design option to stand out while not evoking perceptions of eeriness.

The study was done with great care and consideration, but it was also done during a global pandemic which resulted in the inability to conduct a large-scale laboratory study. Choosing to conduct the study online came with certain drawbacks. The circumstances under which the study was conducted could only be controlled to the degree that participants were asked to put themselves in a distraction-free situation and to scale their screen resolution as described above. Everything else, that is, the screen and keyboard used, the performance of their machine and internet connection and the specifics of their environment might introduce variance with unknown distribution properties to the data. Although the findings are not suspicious of any systematic biases, replicating the experiment under fully controlled laboratory circumstances would yield more robust results.

Moreover, the study is preliminary, considering its intended field of application. The stimuli were presented on a computer screen which was supposedly placed on a desk or in a similar environment. This situation was lacking both the embodiment and the kind of interactivity that would be preferable for an ecologically valid investigation of the GCE in HRI. Studies exploring GCE in an interaction with an actual robot have been done by some researchers (e.g. Kompatsiari et al., 2019; Willemse and Wykowska, 2019) and provided helpful insights. Yet, to the authors’ knowledge none of the studies scrutinized the effect of the stimulus’ sociability. More research along that line is needed to fill the gap between findings from more abstract, fine grained desktop studies, and ecologically more valid studies involving embodied robots and real interaction.

Further, because we aimed at specifically carving out the impact of gaze for joint attention, we presented the stimuli stripped out of their biological context (the face) in a very abstract setting. This enabled to differentiate effects of gaze cueing from other social cues (e.g. head tilt, mimics) and further represented a high ecological validity for the targeted industrial application. However, whether our findings are also valid for more socially embedded applications, for example, the design of humanoid robots, remains an open question for future research.

In sum, the current study provided new insights to the effectiveness and perception of pseudo-social stimuli that can be translated into concrete design recommendations for useful cues fostering joint attention in industrial HRI. The results were overall in favor of the robot crosshair paired eye design. This eye gaze prototype not only performed best in the cueing of social attention, it also received positive ratings on important subjective scales.

## Data Availability

The datasets presented in this study can be found in online repositories. The names of the repository/repositories and accession number(s) can be found below: osf.io/ecta9.

## Footnotes

1 In quotes, because clearly robots do not have intentions. Yet, the intentional stance may be adopted towards artificial agents like robots (Perez-Osorio and Wykowska, 2019).

2 The former studies cited used mere pictures of robots as stimuli.

3 Initially, we also included a fourth item “I think the [stimuli] were controlled consciously.” In hindsight, we ourselves found that question hard to make sense of (for both us and participants) and so dropped it.
